# On the family-free DCJ distance and similarity

**DOI:** 10.1186/s13015-015-0041-9

**Published:** 2015-04-01

**Authors:** Fábio V Martinez, Pedro Feijão, Marília DV Braga, Jens Stoye

**Affiliations:** Faculdade de Computação, Universidade Federal de Mato Grosso do Sul, Avenida Costa e Silva, s-n, Campo Grande, 79070-900 MS Brazil; Technische Fakultät and CeBiTec, Universität Bielefeld, Universitätsstr. 25, Bielefeld, 33615 Germany; Inmetro – Instituto Nacional de Metrologia, Qualidade e Tecnologia, Av. Nossa Senhora das Graças, 50, Duque de Caxias, 25250-020 RJ Brazil

**Keywords:** Genome rearrangement, DCJ, Family-free genome comparison

## Abstract

Structural variation in genomes can be revealed by many (dis)similarity measures. Rearrangement operations, such as the so called double-cut-and-join (DCJ), are large-scale mutations that can create complex changes and produce such variations in genomes. A basic task in comparative genomics is to find the rearrangement distance between two given genomes, i.e., the minimum number of rearragement operations that transform one given genome into another one. In a family-based setting, genes are grouped into gene families and efficient algorithms have already been presented to compute the DCJ distance between two given genomes. In this work we propose the problem of computing the DCJ distance of two given genomes without prior gene family assignment, directly using the pairwise similarities between genes. We prove that this new family-free DCJ distance problem is APX-hard and provide an integer linear program to its solution. We also study a family-free DCJ similarity and prove that its computation is NP-hard.

## Background

Genomes are subject to mutations or rearrangements in the course of evolution. Typical large-scale rearrangements change the number of chromosomes and/or the positions and orientations of genes. Examples of such rearrangements are inversions, translocations, fusions and fissions. A classical problem in comparative genomics is to compute the rearrangement distance, that is, the minimum number of rearrangements required to transform a given genome into another given genome [1].

In order to study this problem, one usually adopts a high-level view of genomes, in which only “relevant” fragments of the DNA (e.g., genes) are taken into consideration. Furthermore, a pre-processing of the data is required, so that we can compare the content of the genomes.

One popular method, adopted for more than 20 years, is to group the genes in both genomes into *gene families*, so that two genes in the same family are said to be equivalent. This setting is said to be *family-based*. Without gene duplications, that is, with the additional restriction that each family occurs exactly once in each genome, many polynomial models have been proposed to compute the genomic distance [2-5]. However, when gene duplications are allowed, the problem is more intrincate and all approaches proposed so far are NP-hard, see for instance [6-10].

It is not always possible to classify each gene unambiguously into a single gene family. Due to this fact, an alternative to the family-based setting was proposed recently and consists in studying the rearrangement distance without prior family assignment. Instead of families, the pairwise similarity between genes is directly used [11,12]. This approach is said to be *family-free*. Although the family-free setting seems to be at least as difficult as the family-based setting with duplications, its complexity is still unknown for various distance models.

In this work we are interested in the problem of computing the distance of two given genomes in a family-free setting, using the *double cut and join* (DCJ) model [5]. The DCJ operation, that consists of cutting a genome in two distinct positions and joining the four resultant open ends in a different way, represents most of large-scale rearrangements that modify genomes. After preliminaries and a formal definition of the family-free DCJ distance, we present a hardness result, before giving a linear programming solution and showing its feasibility for practical problem instances. Finally, we also study the problem of computing the similarity – a counterpart of the distance function – of two given genomes in a family-free setting using the DCJ model and show its NP-hardness.

This paper is an extended version of [13], that was presented at the 14th Workshop on Algorithms in Bioinformatics, WABI 2014.

## Preliminaries

Each gene *g* in a genome is an oriented DNA fragment that can be represented by the symbol *g* itself, if it has direct orientation, or by the symbol −*g*, if it has reverse orientation. Furthermore, each one of the two extremities of a linear chromosome is called a *telomere*, represented by the symbol ∘. Each chromosome in a genome can be represented by a string that can be circular, if the chromosome is circular, or linear and flanked by the symbols ∘ if the chromosome is linear. For the sake of clarity, each chromosome is also flanked by parentheses. As an example, consider the genome *A*={(∘ 3 −1 4 2 ∘),(∘ 5 −6 −7 ∘)} that is composed of two linear chromosomes.

Since a gene *g* has an orientation, we can distinguish its two ends, also called its *extremities*, and denote them by *g*^*t*^ (*tail*) and *g*^*h*^ (*head*). An *adjacency* in a genome is either the extremity of a gene that is adjacent to one of its telomeres, or a pair of consecutive gene extremities in one of its chromosomes. If we consider again the genome *A* above, the adjacencies in its first chromosome are 3^*t*^, 3^*h*^1^*h*^, 1^*t*^4^*t*^, 4^*h*^2^*t*^ and 2^*h*^.

Throughout this paper, let *A* and *B* be two distinct genomes and let  be the set of genes in genome *A* and  be the set of genes in genome *B*.

### Adjacency graph and family-based DCJ distance

**Figure 1:**
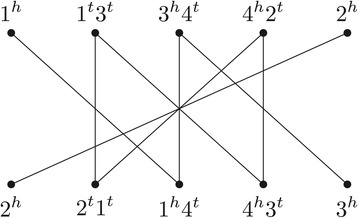
**The adjacency graph for the two unichromosomal and linear genomes** ***A={(∘ −1 3 4 2 ∘)}*** **and** ***B={(∘ −2 1 4 3 ∘)}*** **.**

In the family-based setting the two genomes *A* and *B* have the same content, that is, $\protect \mathcal {A} = \protect \mathcal {B}$. When there are no duplications, that is, when each family is represented by exactly one gene in each genome, the DCJ distance can be easily computed with the help of the *adjacency graphAG*(*A*,*B*), a bipartite multigraph such that each partition corresponds to the set of adjacencies of one of the two input genomes and an edge connects the same extremities of genes in both genomes. In other words, there is a one-to-one correspondence between the set of edges in *AG*(*A*,*B*) and the set of gene extremities. Vertices have degree one or two and thus an adjacency graph is a collection of paths and cycles. An example of an adjacency graph is given in Figure 1.

The family-based DCJ distance d_DCJ_ between two genomes *A* and *B* without duplications can be computed in linear time and is closely related to the number of components in the adjacency graph *AG*(*A*,*B*) [2]:

$$\textup{d}_{\textup{\textsc{dcj}}}(A, B) = n - c - i/2\:, $$

where $n = |\protect \mathcal {A}| = |\protect \mathcal {B}|$ is the number of genes in both genomes, *c* is the number of cycles and *i* is the number of odd paths in *AG*(*A*,*B*).

Observe that, in Figure 1, the number of genes is *n*=4 and *AG*(*A*,*B*) has one cycle and two odd paths. Consequently the DCJ distance is d_DCJ_(*A*,*B*)=4−1−2/2=2.

The formula for d_DCJ_(*A*,*B*) can also be derived using the following approach. Given a component *C* in *AG*(*A*,*B*), let |*C*| denote the length, or number of edges, of *C*. From [14,15] we know that each component in *AG*(*A*,*B*) contributes independently to the DCJ distance, depending uniquely on its length. Formally, the contribution *d*(*C*) of a component *C* in the total distance is given by:

$$d(C) = \left\{ \begin{array}{lll} \frac{|C|}{2} - 1\:, && \text{if \textit{C} is a cycle}\:, \\ \frac{|C|-1}{2}\:, && \text{if \textit{C} is an odd path}\:, \\ \frac{|C|}{2}\:, && \text{if \textit{C} is an even path}\:. \end{array}\right. $$

The sum of the lengths of all components in the adjacency graph is equal to 2*n*. Let , , and  represent the sets of components in *AG*(*A*,*B*) that are cycles, odd paths and even paths, respectively. Then, the DCJ distance can be calculated as the sum of the contributions of each component:

$$\begin{array}{*{20}l} {}\textup{d}_{\textup{\textsc{dcj}}}(A, B) &= \sum_{C \in AG(A, B)}\!\!\!\!d(C) \\ &=\!\! \sum_{C \in \mathcal{C}}\! \left(\!\frac{|C|}{2} \,-\, 1 \!\right) \,+\, \sum_{C \in \mathcal{I}} \left(\frac{|C|-1}{2}\right) + \sum_{C \in \mathcal{P}} \left(\frac{|C|}{2}\right) \\ &= \frac{1}{2} \left(\sum_{C \in AG(A, B)}\!\!\!\!|C| \right) - \sum_{C \in \mathcal{C}} 1 - \sum_{C \in \mathcal{I}}\frac{1}{2} \\ &= n - c - i/2\:. \end{array} $$

### Gene similarity graph for the family-free model

In the family-free setting, each gene in each genome is represented by a distinct symbol, thus $\protect \mathcal {A} \cap \protect \mathcal {B} = \emptyset $ and the cardinalities $|\protect \mathcal {A}|$ and $|\protect \mathcal {B}|$ may be distinct. Let *a* be a gene in *A* and *b* be a gene in *B*, then their *normalized similarity* is given by the value *σ*(*a*,*b*) that ranges in the interval [ 0,1].

**Figure 2:**
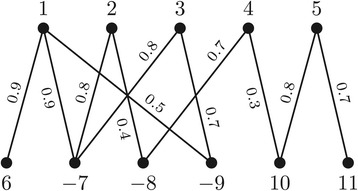
**A possible gene similarity graph for the two unichromosomal linear genomes** ***A={(∘ 1 2 3 4 5 ∘)}*** **and** ***B={(∘ 6 −7 −8 −9 10 11 ∘)}*** **.**

We can represent the similarities between the genes of genome *A* and the genes of genome *B* with respect to *σ* in the so called *gene similarity graph* [12], denoted by *GS*_*σ*_(*A*,*B*). This is a weighted bipartite graph whose partitions  and  are the sets of genes in genomes *A* and *B*, respectively. Furthermore, for each pair of genes (*a*,*b*), such that $a \in \protect \mathcal {A}$ and $b \in \protect \mathcal {B}$, if *σ*(*a*,*b*)>0 there is an edge *e* connecting *a* and *b* in *GS*_*σ*_(*A*,*B*) whose weight is *σ*(*e*):=*σ*(*a*,*b*). An example of a gene similarity graph is given in Figure 2.

## Reduced genomes and their weighted adjacency graph

Let *A* and *B* be two genomes and let *GS*_*σ*_(*A*,*B*) be their gene similarity graph. Now let *M*={*e*_1_,*e*_2_,…,*e*_*n*_} be a matching in *GS*_*σ*_(*A*,*B*) and denote by $w(M) = \sum _{e_{i} \in M} \sigma (e_{i})$ the weight of *M*, that is the sum of its edge weights. Since the endpoints of each edge *e*_*i*_=(*a*,*b*) in *M* are not saturated by any other edge of *M*, we can unambiguously define the function *ℓ*^*M*^(*a*)=*ℓ*^*M*^(*b*)=*i*. The *reduced genomeA*^*M*^ is obtained by deleting from *A* all genes that are not saturated by *M*, and renaming each saturated gene *a* to *ℓ*^*M*^(*a*), preserving its orientation. Similarly, the reduced genome *B*^*M*^ is obtained by deleting from *B* all genes that are not saturated by *M*, and renaming each saturated gene *b* to *ℓ*^*M*^(*b*), preserving its orientation. Observe that the set of genes in *A*^*M*^ and in *B*^*M*^ is $\mathcal {G}(M) = \{ \ell ^{M}(g) \colon g\ \text {is saturated by the matching}\ M \} = \{1,2,\ldots,n\}$.

**Figure 3:**
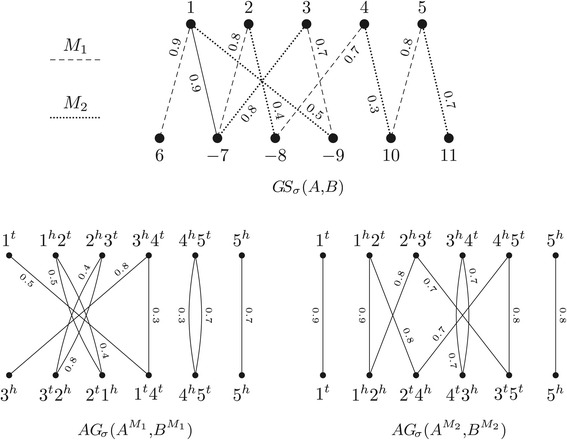
**Reduced genomes and their weighted adjacency graph.** Considering the genomes *A*={(∘ 1 2 3 4 5 ∘)} and *B*={(∘ 6 −7 −8 −9 10 11 ∘)} as in Figure 2, let *M*_1_ (dotted edges) and *M*_2_ (dashed edges) be two distinct matchings in *G* *S*_*σ*_(*A*,*B*), shown in the upper part. The two resulting weighted adjacency graphs $\protect \phantom {\dot {i}\!}{AG}_{\!\sigma }(A^{M_{1}},B^{M_{1}})$, that has two odd paths and three cycles, and $\protect \phantom {\dot {i}\!}{AG}_{\!\sigma }(A^{M_{2}},B^{M_{2}})$, that has two odd paths and two cycles, are shown in the lower part.

Let *A*^*M*^ and *B*^*M*^ be the reduced genomes for a given matching *M* of *GS*_*σ*_(*A*,*B*). The *weighted adjacency graph* of *A*^*M*^ and *B*^*M*^, denoted by *AG*_*σ*_(*A*^*M*^,*B*^*M*^), is obtained by constructing the adjacency graph of *A*^*M*^ and *B*^*M*^ and adding weights to the edges as follows. For each gene *i* in $\protect \mathcal {G}(M)$, both edges *i*^*t*^*i*^*t*^ and *i*^*h*^*i*^*h*^ inherit the weight of edge *e*_*i*_ in *M*, that is, *σ*(*i*^*t*^*i*^*t*^)=*σ*(*i*^*h*^*i*^*h*^)=*σ*(*e*_*i*_). Observe that, for each edge *e*∈*M*, we have two edges of weight *σ*(*e*) in *AG*_*σ*_(*A*^*M*^,*B*^*M*^), thus *w*(*AG*_*σ*_(*A*^*M*^,*B*^*M*^))=2 *w*(*M*) (the weight of *AG*_*σ*_(*A*^*M*^,*B*^*M*^) is twice the weight of *M*). Examples of weighted adjacency graphs are shown in Figure 3.

## The family-free DCJ distance

Based on the weighted adjacency graph, in [12] a family-free DCJ *similarity* measure has been proposed. We will come back to this measure later in this paper. Before that, to be more consistent with the comparative genomics literature, where distance measures are more common than similarities, here we also propose a family-free DCJ *distance*. This family-free distance is based on the weighted DCJ distance of reduced genomes. An important design criterion for this definition is that it must be the same as the (unweighted) family-based DCJ distance when all weights are equal to 1.

The first step in our definition is to consider the components of the graph *AG*_*σ*_(*A*^*M*^,*B*^*M*^) separately, similarly to the approach described previously for the family-based model. Here the contribution of each component *C* is denoted by *d*_*σ*_(*C*) and must include not only the length |*C*| of the component, but also information about the weights of the edges in *C*. Basically, we need a function *f*(*C*) to use instead of |*C*| in the contribution function *d*_*σ*_(*C*), such that: (*i*) when all edges in *C* have weight 1, *f*(*C*)=|*C*|, that is, the contribution of *C* is the same as in the family-based version; (*ii*) when the weights decrease, *f* should increase, because smaller weights mean less similarity, or increased distance between the genomes.

The simplest linear function *f* that satisfies both conditions is *f*(*C*)=2|*C*|−*w*(*C*), where $w(C) = \sum _{e\in C} \sigma (e)$ is the sum of the weights of all the edges in *C*. Then, the *weighted contributiond*_*σ*_(*C*) of the different types of components is:

$$d_{\sigma}(C) = \left\{ \begin{array}{lll} \frac{2|C| - w(C)}{2} - 1\:, && \text{if \textit{C} is a cycle}\:, \\ \frac{2|C| - w(C) -1}{2}\:, && \text{if \textit{C} is an odd path}\:, \\ \frac{2|C| - w(C)}{2}\:, && \text{if \textit{C} is an even path}\:. \end{array}\right. $$

Let , , and  represent the sets of components in *AG*_*σ*_(*A*^*M*^,*B*^*M*^) that are cycles, odd paths and even paths, respectively. Summing the contributions of all the components, the resulting distance for a certain matching *M* is computed as follows:

(1) $$ {\small{\begin{aligned} {} d_{\sigma}(A^{M},B^{M}) & = \sum_{C\in {AG}_{\!\sigma}(A^{M},B^{M})} \!\!\!\!\!d_{\sigma}(C) \\ &=\! \sum_{C \in \mathcal{C}}\!\left(\!\frac{2|C|\,-\,w(C)}{2}-\!1\!\right) \,+\, \sum_{C \in \mathcal{I}}\!\!\left(\!\frac{2|C|\,-\,w(C)\,-\,1}{2}\!\right)\\ &\quad+ \sum_{C \in \mathcal{P}}\!\!\left(\!\frac{2|C|\,-\,w(C)}{2}\!\right) \\ &= \sum_{C \in {AG}_{\!\sigma}(A^{M},B^{M})}\!\!\!\!|C| - \frac{1}{2} \left(\sum_{C \in {AG}_{\!\sigma}(A^{M},B^{M})}\!\!\!\!w(C) \right) \\ &\quad- \sum_{C \in \mathcal{C}} 1 - \sum_{C \in \mathcal{I}}\frac{1}{2} \\ &= 2|M| - w({AG}_{\!\sigma}(A^{M},B^{M}))/2 - c - i/2 \\ &= \textup{d}_{\textup{\textsc{dcj}}}(A^{M}, B^{M}) + |M| - w(M)\:, \end{aligned}}}  $$

since the number of genes in $\protect \mathcal {G}(M)$ is equal to the size of *M*.

Observe that not only the components of the graph, but also the size and the weight of the matching influence the distance above. For example, in Figure 3, matching *M*_1_ gives the weighted adjacency graph with more components, but whose distance $\phantom {\dot {i}\!}d_{\sigma }(A^{M_{1}},B^{M_{1}}) = 1 + 5 - 2.7 = 3.3$ is larger. On the other hand, *M*_2_ gives the weighted adjacency graph with less components, but whose distance $\phantom {\dot {i}\!}d_{\sigma }(A^{M_{2}},B^{M_{2}}) = 2 + 5 - 3.9 = 3.1$ is smaller.

Our goal in the following sections is to study the problem of computing the family-free DCJ distance, i.e., to find a matching in *GS*_*σ*_(*A*,*B*) that minimizes *d*_*σ*_. First of all, it is important to observe that the behaviour of this function does not correlate with the size of the matching. Often smaller matchings, that possibly discard gene assignments, lead to smaller distances. Actually, it is easy to see that, for any pair of genomes with any gene similarity graph, a trivial empty matching leads to the minimum distance, equal to zero. Due to this fact we restrict the distance to *maximal matchings* only. This ensures that no pairs of genes with positive similarity score are simply discarded, even though they might increase the overall distance. Hence we have the following optimization problem:

**Problem**FFDCJ-DISTANCE(*A*,*B*): Given genomes *A* and *B* and their gene similarities *σ*, calculate their family-free DCJ distance

(2) $$ \textup{d}_{\textup{\textsc{ffdcj}}}(A, B) = \min_{M \in \mathbb{M}}\{ d_{\sigma}(A^{M},B^{M}) \} \:,  $$

where  is the set of all maximal matchings in *GS*_*σ*_(*A*,*B*).

## Complexity of the family-free DCJ distance

In order to assess the complexity of FFDCJ-DISTANCE, we use a restricted version of the family-based *exemplar DCJ distance problem* [6,8]:

**Problem** (*s*,*t*)-EXDCJ-DISTANCE(*A*,*B*): Given genomes *A* and *B*, where each family occurs at most *s* times in *A* and at most *t* times in *B*, obtain *exemplar* genomes *A*^′^ and *B*^′^ by removing all but one copy of each family in each genome, so that the DCJ distance d_DCJ_(*A*^′^,*B*^′^) is minimized.

We establish the computational complexity of the FFDCJ-DISTANCE problem by means of a polynomial time and approximation preserving (AP-) reduction from the problem (1,2)-EXDCJ-DISTANCE, which is NP-hard [8]. Note that the authors of [8] only consider unichromosomal genomes, but the reduction can be extended to multichromosomal genomes, since an algorithm that solves the multichromosomal case also solves the unichromosomal case.

### **Theorem1**.

Problem FFDCJ-DISTANCE(*A*,*B*) is APX-hard, even if the maximum degrees in the two partitions of *GS*_*σ*_(*A*,*B*) are respectively one and two.

Before proving the result, we need some definitions and particularly a formal definition of an AP-reduction. These definitions are based on [16].

An *optimization problem* is defined by three main elements: a set of instances, a set Sol(*I*) of *feasible solutions* for each instance *I*, and a function val that relates a non-negative rational number val(*I*,*S*) to each instance *I* and solution *S* in Sol(*I*). Thus, in a minimization problem, the aim is to find a feasible solution of minimum value. That is, if *Π* is an optimization problem with an instance *I*, then we want to find *S*∈Sol(*I*) that minimizes val(*I*,*S*), called an *optimal solution* to the optimization problem. For an instance *I*, the value of an optimal solution is denoted by opt(*I*).

An *AP-reduction* from an optimization problem *Π* to an optimization problem *Π*^′^ is a triple (*f*,*g*,*β*), where *f* and *g* are algorithms and *β* is a positive rational number, such that:

- *f* receives as input a positive rational number *δ* and an instance *I* of *Π*, and returns an instance *f*(*δ*,*I*) of *Π*^′^;
- *g* receives as input a positive rational number *δ*, an instance *I* of *Π* and an element *S*^′^ in Sol(*f*(*δ*,*I*)), and returns a solution *g*(*δ*,*I*,*S*^′^) in Sol(*I*);
- for any positive rational number *δ*, *f*(*δ*,·) and *g*(*δ*,·,·) are polynomial time algorithms;
- for any instance *I* of *Π*, any positive rational number *δ*, and any *S*^′^ in Sol(*f*(*δ*,*I*)), if
  $$ \text{val}(f(\delta, I), S') \leq (1 + \delta) \: \text{opt}(f(\delta, I))\:, $$
  then
  $$ \text{val}(I, g(\delta, I, S')) \leq (1 + \beta\delta) \, \text{opt}(I)\:. $$

An AP-reduction from *Π* to *Π*^′^ is frequently denoted by *Π*≤_AP_*Π*^′^, and we say that *Π* is *AP-reduced* to *Π*^′^. An AP-reduction is a special type of reduction which preserves both the polynomiality property and the approximation factor.

Now, we can proceed with the proof of Theorem 1.

### Proof 1.

**Figure 4:**
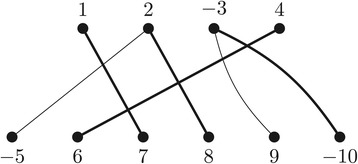
**Gene similarity graph** ***GS***_***σ***_ ***(A***_***F***_ ***,B***_***F***_ ***)*** **constructed from the input genomes** ***A={(∘ a c −b d ∘)}*** **and** ***B={(∘ −c d a c b −b ∘)}*** **of** ***(1,2)*** **-** EXDCJ-DISTANCE **, where all edge weights are 1.** Highlighted edges represent a maximal matching *M* in *G* *S*_*σ*_(*A*_F_,*B*_F_).

(of Theorem 1). We give an AP-reduction (*f*,*g*,*β*) from (1,2)-EXDCJ-DISTANCE to FFDCJ-DISTANCE.

(AP1) Algorithm *f* receives as input a positive rational number *δ* and an instance (*A*,*B*) of (1,2)-EXDCJ-DISTANCE where *A* and *B* are genomes from a set of genes  and each gene in  occurs at most once in *A* and at most twice in *B*, and constructs an instance (*A*_F_,*B*_F_)=*f*(*δ*,(*A*,*B*)) of FFDCJ-DISTANCE as follows. Let the genes of *A* be denoted *a*_1_,*a*_2_,…,*a*_|*A*|_ and the genes of *B* be denoted *b*_1_,*b*_2_,…,*b*_|*B*|_. Then *A*_F_ and *B*_F_ are copies of *A* and *B*, respectively, except that symbol *a*_*i*_ in *A*_F_ is relabeled by *i*, keeping its orientation, and *b*_*j*_ in *B*_F_ is relabeled by *j*+|*A*|, also keeping its orientation. Furthermore, the normalized similarity measure *σ* for genes in *A*_F_ and *B*_F_ is defined as *σ*(*i*,*k*)=1 for *i* in *A*_F_ and *k* in *B*_F_, such that *a*_*i*_ is in *A*, *b*_*j*_ is in *B*, *a*_*i*_ and *b*_*j*_ are in the same gene family, and *k*=*j*+|*A*|. Otherwise, *σ*(*i*,*k*)=0. Note that the construction is independent of the value of *δ*. Figure 4 refers to an example of a gene similarity graph *GS*_*σ*_(*A*_F_,*B*_F_) of this construction.

(AP2) Algorithm *g* receives as input a positive rational number *δ*, an instance (*A*,*B*) of (1,2)-EXDCJ-DISTANCE and a solution *M* of FFDCJ-DISTANCE, and transforms *M* into a solution (*A*_X_,*B*_X_) of (1,2)-EXDCJ-DISTANCE. This is a simple construction: for each edge (*i*,*k*) in *M*, we add symbols *a*_*i*_ to *A*_X_ and *b*_*j*_ to *B*_X_, where *j*=*k*−|*A*|. The value of *δ* does not influence the construction. In the example of Figure 4, a matching *M*={(1,7),(2,8),(−3,−10),(4,6)}, which is a solution to FFDCJ-DISTANCE (*A*_F_,*B*_F_), is transformed by *g* into the genomes *A*_*X*_={(∘ *a*_1_*a*_2_*a*_3_*a*_4_ ∘)}={(∘ *ac* −*bd* ∘)} and *B*_*X*_={(∘ *b*_2_*b*_3_*b*_4_*b*_6_ ∘)}={(∘ *dac* −*b* ∘)}, which is a solution to (1,2)-EXDCJ-DISTANCE (*A*,*B*).

(AP3) Clearly, for any positive rational number *δ*, functions *f* and *g* are polynomial time algorithms on the size of their respective instances. A schematic view of these transformations is presented below.

$$\begin{array}{ccc} \textsc{(1,2)-exdcj-distance} & & \textsc{ffdcj-distance} \\[.3cm] (A, B) & \:\: \xrightarrow{f, \delta} \:\: & (A_{\mathrm{F}}, B_{\mathrm{F}}) := f(\delta, (A, B)) \\ \left\downarrow\rule{0cm}{.4cm}\right. & &\left\downarrow\rule{0cm}{.4cm}\right. \\ (A_{\textsc{x}}, B_{\textsc{x}}) := g(\delta, (A, B), M) &\xleftarrow{g, \delta} & M \end{array} $$

(AP4) Finally, suppose that for an instance (*A*,*B*)of (1,2)-EXDCJ-DISTANCE, a positive rational number *δ*and a solution *M* of FFDCJ-DISTANCE with instance (*A*_F_,*B*_F_)=*f*(*δ*,(*A*,*B*)), we have

$$d_{\sigma}(A_{\mathrm{F}}^{M}, B_{\mathrm{F}}^{M}) \leq (1 + \delta) \, \text{opt}(\textsc{ffdcj-distance}(A_{\mathrm{F}}, B_{\mathrm{F}}))\:. $$

Let *A*_X_:=*A* and *B*_X_ be an exemplar genome of *B*, such that (*A*_X_,*B*_X_)=*g*(*δ*,(*A*,*B*),*M*). We want to prove that (*A*_X_,*B*_X_) is such that

(3) $$  {}d(A_{\textsc{x}}, B_{\textsc{x}}) \leq (1 + \beta\delta) \, \text{opt}(\textsc{(1,2)-exdcj-distance}(A, B))  $$

for some fixed positive rational number *β*.

Denote by *c*_*AG*_ and *i*_*AG*_ the number of cycles and odd paths, respectively, in the adjacency graph *AG*(*A*_X_,*B*_X_), and by $\phantom {\dot {i}\!}c_{{AG}_{\!\sigma }}$ and $\phantom {\dot {i}\!}i_{{AG}_{\!\sigma }}$ the number of cycles and odd paths, respectively, in the weighted adjacency graph ${AG}_{\!\sigma }(A_{\mathrm {F}}^{M}, B_{\mathrm {F}}^{M})$.

Observe that the way the functions *f* and *g* have been defined, we have |*A*_X_|=|*B*_X_|=|*M*|, $\phantom {\dot {i}\!}c_{\textit {AG}} = c_{{AG}_{\!\sigma }}$, $\phantom {\dot {i}\!}i_{\textit {AG}} = i_{{AG}_{\!\sigma }}$, and thus

$$\begin{array}{*{20}l} d_{\sigma}(A_{\mathrm{F}}, B_{\mathrm{F}}) &= 2|M| - w(M) - c_{{AG}_{\!\sigma}} - i_{{AG}_{\!\sigma}} / 2 \\ &= 2|M| - |M| - c_{{AG}_{\!\sigma}} - i_{{AG}_{\!\sigma}} / 2 \\ &= |M| - c_{{AG}_{\!\sigma}} - i_{{AG}_{\!\sigma}} / 2 \\ &= |A_{\textsc{x}}| - c_{AG} - i_{AG} / 2 \\ &= d(A_{\textsc{x}}, B_{\textsc{x}}) \:. \end{array} $$

Particularly, it is easy to see that we have

$$\begin{aligned} \text{opt}&(\textsc{ffdcj-distance}(A_{\mathrm{F}}, B_{\mathrm{F}})) \\ &=\text{opt}(\textsc{(1,2)-exdcj-distance}(A, B)) \:. \end{aligned} $$

Therefore,

$$\begin{aligned} {}d(A_{\textsc{x}}, B_{\textsc{x}}) &= d_{\sigma}(A_{\mathrm{F}}, B_{\mathrm{F}}) \\ &\leq (1+ \delta) \, \text{opt}(\textsc{ffdcj-distance}(A_{\mathrm{F}}, B_{\mathrm{F}})) \\ &= (1 + \delta) \, \text{opt}(\textsc{(1,2)-exdcj-distance}(A, B))\,, \end{aligned} $$

and Equation (3) holds by setting *β*:=1.

### **Corollary2**.

There exists no polynomial-time algorithm for FFDCJ-DISTANCE with approximation factor better than 1237/1236, unless P = NP.

### *Proof*.

As shown in [8], (1,2)-EXDCJ-DISTANCE is NP-hard to approximate within a factor of 1237/1236−*ε* for any *ε*>0. Therefore, the result follows immediately from [8] and from the AP-reduction in the proof of Theorem 1.

Since the weight plays an important role in *d*_*σ*_, a matching with maximum weight, that is obviously maximal, could be a candidate for the design of an approximation algorithm for FFDCJ-DISTANCE. However, we can demonstrate that it is not possible to obtain such an approximation, with the following example.

Consider an integer *k*≥1 and let *A*={(∘ 1 −2 ⋯ (2*k* − 1) −2*k* ∘)} and *B*={(∘ −(2*k* + 1) (2*k* + 2) ⋯ −(2*k* + 2*k* − 1) (2*k* + 2*k*) ∘)} be two unichromosomal linear genomes. Observe that *A* and *B* have an even number of genes with alternating orientation. While *A* starts with a gene in direct orientation, *B* starts with a gene in reverse orientation. Now let *σ* be the normalized similarity measure between the genes of *A* and *B*, defined as follows:

$${\fontsize{7.1}{6}\begin{aligned} ~\sigma(i, j) = \left\{ \begin{array}{ll} 1\:\!, & \text{for each} ~i \in \{1,2,\ldots,2k\} \text{ and } j\,=\,2k\,+\,i\:; \\ 1\,-\,\varepsilon\:\!, & \text{for each}~ i \in \{1,3,\ldots,2k\,-\,1\} \text{ and } j\,=\,2k\,+\,i\,+\,1, \text{with }\varepsilon \in [0, 1)\:\!;\\ 0\:\!, & \text{otherwise}\:\!.\\ \end{array} \right. \end{aligned}} $$

**Figure 5:**
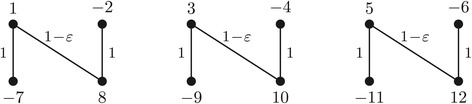
**Gene similarity graph** ***GS***_***σ***_ ***(A,B)*** **for** ***k=3*** **.**

Figure 5 shows *GS*_*σ*_(*A*,*B*) for *k*=3 and *σ* as defined above.

There are several matchings in *GS*_*σ*_(*A*,*B*). We are interested in two particular maximal matchings:

- *M*^∗^ is composed of all edges that have weight 1−*ε*. It has weight *w*(*M*^∗^)=(1−*ε*)|*M*^∗^|=(1−*ε*) *k*/2. Its corresponding weighted adjacency graph $\phantom {\dot {i}\!}{AG}_{\!\sigma }(A^{M^{*}}\!,B^{M^{*}})$ has |*M*^∗^|−1 cycles and two odd paths, thus $\text {d}_{\text {\textsc {dcj}}}(A^{M^{*}}\!\!,B^{M^{*}})=0$. Consequently, we have $\phantom {\dot {i}\!}d_{\sigma }(A^{M^{*}}\!\!,B^{M^{*}}) =|M^{*}|- (1-\varepsilon)|M^{*}|=\varepsilon |M^{*}|$.
- *M* is composed of all edges that have weight 1. It is the only matching with the maximum weight *w*(*M*)=|*M*|=*k*. Its corresponding weighted adjacency graph *AG*_*σ*_(*A*^*M*^,*B*^*M*^) has two even paths, but no cycles or odd paths, giving d_dcj_(*A*^*M*^,*B*^*M*^)=|*M*|. Hence, *d*_*σ*_(*A*^*M*^,*B*^*M*^)=2|*M*|−|*M*|=|*M*|.

Notice that $\text {d}_{\text {\textsc {ffdcj}}}(A,B)\leq {d}_{\sigma }(A^{M^{*}},B^{M^{*}})$. Furthermore, since |*M*|=2|*M*^∗^|,

$$\frac{d_{\sigma}(A^{M},B^{M})}{d_{\sigma}(A^{M^{*}},B^{M^{*}})} = \frac{|M|}{\varepsilon|M^{*}|} = \frac{k}{\varepsilon \, k/2} = \frac{2}{\varepsilon} $$

and 2/*ε*→+*∞* when *ε*→0.

This shows that, for any genomes *A* and *B*, a matching of maximum weight in *GS*_*σ*_(*A*,*B*) can have *d*_*σ*_ arbitrarily far from the optimal solution and cannot give an approximation for FFDCJ-DISTANCE(*A*,*B*).

## ILP to compute the family-free DCJ distance

We propose an integer linear program (ILP) formulation to compute the family-free DCJ distance between two given genomes. This formulation is a slightly different version of the ILP for the maximum cycle decomposition problem given by Shao *et al.* [10] to compute the DCJ distance between two given genomes with duplicate genes. Besides the cycle decomposition in a graph, as was made in [10], we also have to take into account maximal matchings in the gene similarity graph and their weights.

Let *A* and *B* be two genomes with extremity sets *X*_*A*_ and *X*_*B*_, respectively, and let *G*=*GS*_*σ*_(*A*,*B*) be their gene similarity graph. The weight *w*(*e*) of an edge *e* in *G* is also denoted by *w*_*e*_. Let *M* be a maximal matching in *G*. For the ILP formulation, a weighted adjacency graph *H*=*AG*_*σ*_(*A*^*M*^,*B*^*M*^) is such that *V*(*H*)=*X*_*A*_∪*X*_*B*_ and *E*(*H*) has three types of edges: (*i*) *matching edges* that connect two extremities in different extremity sets, one in *X*_*A*_ and the other in *X*_*B*_, if there exists one edge in *M* connecting these genes in *G*; the set of matching edges is denoted by *E*_*m*_; (*ii*) *adjacency edges* that connect two extremities in the same extremity set if they are an adjacency; the set of adjacency edges is denoted by *E*_*a*_; and (*iii*) *self edges* that connect two extremities of the same gene in an extremity set; the set of self edges is denoted by *E*_*s*_. All edges in *H* are in *E*_*m*_∪*E*_*a*_∪*E*_*s*_=*E*(*H*). Matching edges have weights defined by the normalized similarity *σ*, all adjacency edges have weight 1, and all self edges have weight 0. Notice that any edge in *G* corresponds to two matching edges in *H*.

Now we describe the ILP. For each edge *e* in *H*, we create the binary variable *x*_*e*_ to indicate whether *e* will be in the final solution. We require first that each adjacency edge be chosen:

$$x_{e} = 1\:, \qquad \forall~e \in E_{a}\:. $$

We require then that, for each vertex in *H*, exactly one incident edge to it be chosen:

$${\small{\begin{aligned} {} \sum_{uv \in E_{m} \cup E_{s}} x_{uv} \,=\, 1\:,\: \forall~u \in X_{A}\:, \;\, \text{and} \;\, \sum_{uv \in E_{m} \cup E_{s}} x_{uv} \,=\, 1\:, \: \forall~v \in X_{B}\:. \end{aligned}}} $$

Then, we require that the final solution be consistent, meaning that if one extremity of a gene in *A* is assigned to an extremity of a gene in *B*, then the other extremities of these two genes have to be assigned as well:

$$x_{a^{h}b^{h}} = x_{a^{t}b^{t}}\:, \qquad \forall~ab \in E(G)\:. $$

We also require that the matching be maximal. This can easily be ensured if we guarantee that at least one of the vertices connected by an edge in the gene similarity graph be chosen, which is equivalent to not allowing both of the corresponding self edges in the weighted adjacency graph be chosen:

$$x_{a^{h}a^{t}} + x_{b^{h}b^{t}} \leq 1\:, \qquad \forall~ab \in E(G)\:. $$

To count the number of cycles, we use the same strategy as described in [10]. We first give an arbitrary index for each vertex in *H* such that *V*(*H*)={*v*_1_,*v*_2_,…,*v*_*k*_} with *k*=|*V*(*H*)|. For each vertex *v*_*i*_, we define a variable *y*_*i*_ that labels *v*_*i*_ such that

$$0 \leq y_{i} \leq i\:, \qquad 1 \leq i \leq k\:. $$

We also require that adjacent vertices have the same label, forcing all vertices in the same cycle to have the same label:

$$\begin{array}{@{}rcl@{}} y_{i} \leq y_{j} + i \cdot (1 - x_{e})\:, & & \forall~e = v_{i}v_{j} \in E(H)\:, \\ y_{j} \leq y_{i} + j \cdot (1 - x_{e})\:, & & \forall~e = v_{i}v_{j} \in E(H)\:. \end{array} $$

We create a binary variable *z*_*i*_, for each vertex *v*_*i*_, to verify whether *y*_*i*_ is equal to its upper bound *i*:

$$i\cdot z_{i} \leq y_{i}\:, \qquad 1 \leq i \leq k\:. $$

Since all the *y*_*i*_ variables in the same cycle have the same label but a different upper bound, only one of the *y*_*i*_ can be equal to its upper bound *i*. This means that for each cycle there can be only one *z*_*i*_ equal to 1, and the sum of all *z*_*i*_ variables is the total number of cycles in the adjacency graph.

In fact, it is possible to reduce the number of *z*_*i*_ variables. First, notice that each cycle always has vertices from both genomes. That means that if we label all vertices *v*_*i*_ starting with vertices of genome *A* first and then genome *B*, then the upper bounds for all *y*_*i*_s from genome *A* are smaller than the upper bounds for the *y*_*i*_s from genome *B*, and therefore no *z*_*i*_ from genome *B* will ever be 1, since in the same cycle there will be at least one *y*_*i*_ from genome *A* with a smaller upper bound. Then, all *z*_*i*_ corresponding to vertices of genome *B* may be discarded:

$$i\cdot z_{i} \leq y_{i}\:, \qquad 1 \leq i \leq |X_{A}| \:. $$

Finally, we set the objective function as follows:

$$\text{minimize} \quad 2\sum_{e \in E_{m}} x_{e} - \sum_{e \in E_{m}} w_{e}x_{e} - \sum_{1 \leq i \leq |X_{A}|} z_{i}\:, $$

which is exactly the family-free DCJ distance d_FFDCJ_(*A*,*B*) as defined in Equations (1) and (2).

### Simulations and experimental results

We performed some initial benchmarking experiments of the proposed ILP formulation. Therefore, we produced datasets using the Artificial Life Simulator (ALF) [17]. Genome sizes varied from 1000 to 3000 genes, where the gene lengths were generated according to a gamma distribution with shape parameter *k*=3 and scale parameter *θ*=133. A birth-death tree with 10 leaves was generated, with PAM distance of 100 from the root to the deepest leaf. For the amino acid evolution, the WAG substitution model with default parameters was used, with Zipfian indels at a rate of 0.000005. For structural evolution, gene duplications and gene losses were applied with a rate of 0.001 and reversals and translocations with a rate of 0.0025. To test different proportions of rearrangement events, we also simulated datasets where the structural evolution ratios had a 2- and 5-fold increase.

To solve the ILPs, we ran the CPLEX Optimizer on the 45 pairwise comparisons of each simulated dataset. All simulations were run in parallel on a cluster consisting of machines with an Intel(R) Xeon(R) E7540 CPU, with 48 cores and as many as 2 TB of memory, but for each individual CPLEX run only 4 cores and 2 GB of memory were allocated. The results are summarized in Table 1.

**Table 1:** **ILP running-time results for datasets with different genome sizes and evolutionary rates**

	**1000 genes**				**2000 genes**				**3000 genes**		
	***r*** **=1**	***r*** **=2**	***r*** **=5**		***r*** **=1**	***r*** **=2**	***r*** **=5**		***r*** **=1**	***r*** **=2**	***r*** **=5**
Finished	35/45	10/45	2/45		45/45	9/45	1/45		45/45	7/45	3/45
Avg. Time (s)	99.66	6.97	0.53		0.47	0.70	3.31		0.45	2.03	213.15
Avg. Gap (%)	0.3	3.0	4.3		0	3.6	6.5		0	5.3	4.8

Each dataset has 10 genomes, totalling 45 pairwise comparisons. Maximum running time was set to 60 minutes. For each dataset, the number of runs is shown that found an optimal solution within the allowed time and their average running time in seconds. For the runs that did not finish, the last row shows the relative gap between the upper bound and the current solution. Rate *r*=1 means the default rate for ALF evolution, and *r*=2 and *r*=5 mean 2-fold and 5-fold increase for the gene duplication, gene deletion and rearrangement rates.

## The family-free DCJ similarity

For a given matching *M* in *GS*_*σ*_(*A*,*B*), a formula for the similarity *s*_*σ*_ of the reduced genomes *A*^*M*^ and *B*^*M*^ was first proposed in [12] only considering the cycles of *AG*_*σ*_(*A*^*M*^,*B*^*M*^). Here we extend this formula to consider all components of the weighted adjacency graph. Again, let , , and  represent the sets of components in *AG*_*σ*_(*A*^*M*^,*B*^*M*^) that are cycles, odd paths and even paths, respectively. Furthermore, $w(C) = \sum _{e\in C} \sigma (e)$ is the sum of the weights of all the edges in a component *C*. Then the similarity *s*_*σ*_ is the normalized total weight of all components:

$${}s_{\sigma}(A^{M},B^{M}) \,=\,\! \sum_{C \in \mathcal{C}}\!\left(\!\frac{w(C)}{|C|}\!\right) +\!\sum_{C \in \mathcal{I}}\!\left(\! \frac{w(C)}{|C|\,+\,1}\!\right) +\!\!\sum_{C \in \mathcal{P}}\!\left(\!\frac{w(C)}{|C|\,+\,2}\!\right)\:.$$

Here our goal is to study the problem of computing the family-free DCJ similarity, i.e., to find a matching in *GS*_*σ*_(*A*,*B*) that maximizes *s*_*σ*_. Similarly to the distance, the behaviour of the similarity does not correlate with the size of the matching. In other words, smaller matchings, that possibly discard gene assignments, can lead to higher similarities.

An approach for solving this problem was proposed in [12], following the one in [11] for gene adjacencies. It consists of a parameterized similarity function _*α*_ in which the user-controlled parameter *α* is a real number between 0 and 1:

$${\cal F}_{\alpha}(A^{M},B^{M}) = \alpha \cdot s_{\sigma}(A^{M},B^{M}) + (1-\alpha) \cdot w(M)\:, $$

where, as above, $w(M) = \sum _{e \in M} w(e)$ is the sum of the edge weights of the matching *M*.

Observe that the parameter *α* can be adjusted in favor of gene similarity when *α* is closer to 0, or in favor of genome organization similarity, when *α* is closer to 1. The closer the parameter *α* is to 0, the closer we are to the problem of finding a maximum weighted matching in the gene similarity graph *GS*_*σ*_(*A*,*B*). On the other hand, the closer *α* is to 1, the closer we are to the problem of computing *s*_*σ*_(*A*^*M*^,*B*^*M*^). A drawback of this model is that the weights of edges actually appear in both terms of the equation. Furthermore, it remains the problem of finding the “best” value for *α*.

Here, instead of adopting the parameter *α*, we restrict the similarity to *maximal matchings* only, ensuring that no pair of genes with positive similarity score is simply discarded, even though it might decrease the overall similarity. We then have the following optimization problem:

**Problem**FFDCJ-SIMILARITY(*A*,*B*): Given genomes *A* and *B* and their gene similarities *σ*, calculate their family-free DCJ similarity

$$\text{s}_{\text{\textsc{ffdcj}}}(A, B) = \max_{M \in \mathbb{M}}\{ s_{\sigma}(A^{M},B^{M}) \} \:, $$

where  is the set of all maximal matchings in *GS*_*σ*_(*A*,*B*).

### Complexity of the family-free DCJ similarity

We have the following result to the family-free DCJ similarity.

#### **Theorem3**.

Problem FFDCJ-SIMILARITY is NP-hard, even if the maximum degrees in the two partitions of the gene similarity graph are respectively one and two.

#### *Proof*.

We use the Cook reduction, which is a polynomial time transformation, from (1,2)-EXDCJ-DISTANCE to FFDCJ-SIMILARITY.

Let *A* and *B* be any instance of (1,2)-EXDCJ-DISTANCE and let *k* be a positive integer, with *k*≤|*A*|, where |*A*| is the number of genes of a genome *A*. We suppose, without loss of generality, that *A* and *B* are circular multichromosomal genomes. We must construct a pair of circular genomes *A*_F_ and *B*_F_, a normalized similarity measure *σ* for genes in *A*_F_ and *B*_F_, and a positive integer *k*^′^≤|*A*_F_| such that the family-free DCJ similarity of *A*_F_ and *B*_F_ is at least *k*^′^ if and only if the exemplar DCJ distance of genomes *A* and *B* is at most *k*.

The construction of *A*_F_,*B*_F_,*σ*, and *k*^′^ is similar to the transformation *f* in (AP1) of the proof of Theorem 1. Let  be the underlying gene set, such that each gene in  occurs at most once in *A* and at most twice in *B*. Let the genes of *A* be denoted *a*_1_,*a*_2_,…,*a*_|*A*|_ and the genes of *B* be denoted *b*_1_,*b*_2_,…,*b*_|*B*|_. Then *A*_F_ and *B*_F_ are copies of *A* and *B*, respectively, except that symbol *a*_*i*_ in *A*_F_ is relabeled by *i*, keeping its orientation, and *b*_*j*_ in *B*_F_ is relabeled by *j*+|*A*|, also keeping its orientation. The normalized similarity measure *σ* for genes in *A*_F_ and *B*_F_ is defined as *σ*(*i*,*k*)=1 for *i* in *A*_F_ and *k* in *B*_F_, such that *a*_*i*_ is in *A*, *b*_*j*_ is in *B*, *a*_*i*_ and *b*_*j*_ are in the same gene family, and *k*=*j*+|*A*|. Otherwise, *σ*(*i*,*k*)=0. It is easy to see that this construction can be accomplished in poynomial time.

Now we must show that the family-free DCJ similarity of *A*_F_ and *B*_F_ is at least *k*^′^ if and only if the exemplar DCJ distance of genomes *A* and *B* is at most *k*. Let *n*=|*A*|.

Suppose first that *M* is a matching in the gene similarity graph *GS*_*σ*_(*A*_F_,*B*_F_) such that $s_{\sigma }(A_{\mathrm {F}}^{M}, B_{\mathrm {F}}^{M}) \geq k'$. For each edge (*i*,*k*) in *M*, we add symbols *a*_*i*_ to *A*_X_ and *b*_*j*_ to *B*_X_, where *j*=*k*−|*A*|. Notice that $|M| = |A_{\mathrm {F}}^{M}| = |A_{\textsc {x}}| = |A|$. Then, since the genomes in both instances are circular and the edge weights in the gene similarity graph of *A*_F_ and *B*_F_ are all one, we have

$${\fontsize{8.6pt}{9.6pt}\selectfont{ \begin{aligned} {} k'\leq s_{\sigma}(A_{\mathrm{F}}^{M}, B_{\mathrm{F}}^{M}) = \sum_{C \in \mathcal{C}} \frac{w(C)}{|C|} = c_{{AG}_{\!\sigma}} = c_{AG} = |M| - d(A_{\textsc{x}}, B_{\textsc{x}}) \:, \end{aligned}}} $$

where *c*_*AG*_ is the number of cycles in the adjacency graph *AG*(*A*_X_,*B*_X_). Thus, by setting *k*=*n*−*k*^′^, we have

$$d(A_{\textsc{x}}, B_{\textsc{x}}) \leq n - k' = k\:. $$

On the other hand, suppose that for an instance (*A*,*B*) of (1,2)-EXDCJ-DISTANCE we have exemplar genomes *A*_X_ and *B*_X_ such that *d*(*A*_X_,*B*_X_)≤*k*. The exemplar genomes *A*_X_ and *B*_X_ induce a matching *M* in the gene similarity graph *GS*_*σ*_(*A*_F_,*B*_F_) and, once again, since the genomes in both instances are circular and the edge weights in *GS*_*σ*_(*A*_F_,*B*_F_) are all one, we have

$$ \begin{aligned} {}k \geq d(A_{\textsc{x}}, B_{\textsc{x}}) &= n - c_{AG} = n - c_{{AG}_{\!\sigma}} = n - \sum_{C \in\,\mathcal{C}} \frac{w(C)}{|C|} \\ &= n - s_{\sigma}\left(A_{\mathrm{F}}^{M}, B_{\mathrm{F}}^{M}\right)\:, \end{aligned}  $$

where *c*_*AG*_ is the number of cycles in the adjacency graph *AG*(*A*_X_,*B*_X_). By setting *k*^′^=*n*−*k* we have

$$s_{\sigma}(A_{\mathrm{F}}^{M}, B_{\mathrm{F}}^{M}) \geq n - k = k'\:. $$

## Conclusion

In this paper, we have defined a new distance measure for two genomes that is motivated by the double cut and join model, while not relying on gene annotations in form of gene families. In case gene families are known and each family has exactly one member in each of the two genomes, this distance equals the family-based DCJ distance and thus can be computed in linear time. In the general case, however, it is NP-hard and even hard to approximate. Nevertheless, we could give an integer linear program for the exact computation of the distance that is fast enough to be applied to realistic problem instances. Similar theoretical results hold for the family-free DCJ similarity measure, which is NP-hard.

The family-free model has many potentials when gene family assignments are not available or ambiguous, in fact it can even be used to improve family assignments [18]. The work presented in this paper is another step in this direction.
